# Value of adenosine infusion for infarct size determination using real-time myocardial contrast echocardiography

**DOI:** 10.1186/1476-7120-4-10

**Published:** 2006-02-08

**Authors:** Paulo Magno Martins Dourado, Jeane Mike Tsutsui, Antonio Carlos Palandri Chagas, João César Nunes Sbano, Vera demarchi Aiello, Protásio Lemos da Luz, Wilson Mathias Jr, Jose AF Ramires

**Affiliations:** 1 Heart Institute (InCor), University of São Paulo Medical School, São Paulo, Brazil

## Abstract

**Background:**

Myocardial contrast echocardiography has been used for determination of infarct size (IS) in experimental models. However, with intermittent harmonic imaging, IS seems to be underestimated immediately after reperfusion due to areas with preserved, yet dysfunctional, microvasculature. The use of exogenous vasodilators showed to be useful to unmask these infarcted areas with depressed coronary flow reserve. This study was undertaken to assess the value of adenosine for IS determination in an open-chest canine model of coronary occlusion and reperfusion, using real-time myocardial contrast echocardiography (RTMCE).

**Methods:**

Nine dogs underwent 180 minutes of coronary occlusion followed by reperfusion. PESDA (Perfluorocarbon-Exposed Sonicated Dextrose Albumin) was used as contrast agent. IS was determined by RTMCE before and during adenosine infusion at a rate of 140 mcg·Kg^-1^·min^-1^. Post-mortem necrotic area was determined by triphenyl-tetrazolium chloride (TTC) staining.

**Results:**

IS determined by RTMCE was 1.98 ± 1.30 cm^2 ^and increased to 2.58 ± 1.53 cm^2 ^during adenosine infusion (p = 0.004), with good correlation between measurements (r = 0.91; p < 0.01). The necrotic area determined by TTC was 2.29 ± 1.36 cm^2 ^and showed no significant difference with IS determined by RTMCE before or during hyperemia. A slight better correlation between RTMCE and TTC measurements was observed during adenosine (r = 0.99; p < 0.001) then before it (r = 0.92; p = 0.0013).

**Conclusion:**

RTMCE can accurately determine IS in immediate period after acute myocardial infarction. Adenosine infusion results in a slight better detection of actual size of myocardial damage.

## Background

IS can be determined by myocardial contrast echocardiography after a period of coronary ischemia and reperfusion, by exploiting the "no-reflow" state of necrotic tissue [1-4]. In this setting, fewer microbubbles enter the necrotic zone because of functional and/or structural damage to the coronary capillaries, resulting in a hypoperfused region that can be detected by this non-invasive method [5]. However, previous studies using intermittent harmonic imaging demonstrated that IS can be underestimated by myocardial contrast echocardiography immediately after coronary reperfusion, because in some regions the microvascular damage occurs later than myocyte death, resulting in relatively preserved perfusion in infarcted areas [6-8]. In this setting, exogenous vasodilatation has proven to be useful to unmask necrotic area with abnormal microvascular coronary flow reserve, providing a more accurate assessment of IS.

RTMCE is a technique that uses very low mechanical energy and, thus, allows the observation of real-time myocardial perfusion as a result of reduced background-tissue interference. Therefore, it is possibly the ideal tool for the evaluation of dynamic changes that occur in coronary blood flow and simultaneous evaluation of myocardial function [9]. This study was undertaken to assess the additional value of exogenous vasodilation with adenosine for IS determination using RTMCE, in an open-chest canine model of coronary occlusion and reperfusion.

## Methods

### Animal preparation

The protocol was conformed to the American Heart Association Guidelines for Animal Research Use. Twelve mongrel dogs weighting 12 to 20 Kg were anesthetized with sodium pentobarbital (30 mg·kg^-1 ^body weight), intubated and mechanically ventilated. Additional anesthesia was administered during the experiment as needed. A 7F catheter was positioned in the left carotid artery for monitoring systemic arterial pressure, connected to a Biopac Systems device (Biopac Systems, California), and another 7F catheter was introduced into the jugular vein for drug infusions and volemic control.

A left lateral thoracotomy was performed, and the heart suspended in a pericardial cradle. The proximal left anterior descending coronary artery (LAD) was then dissected free from the surrounding tissue, and, at the time of coronary occlusion, an occluder was placed around of it. A two millimeter transit-time ultrasound probe (Transonic Systems, Inc, Probe 2SB839) was placed around the LAD, proximal to the occluder, and connected to a digital flowmeter for epicardial coronary flow measurements.

### Myocardial contrast echocardiography

**Figure 1:**
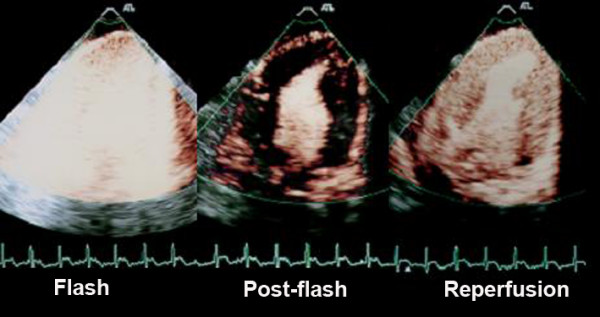
Example of a baseline representative study showing real-time myocardial contrast echocardiography with flash imaging used to cause myocardial microbubble destruction on the **left**, followed by absence of myocardial contrast in post-flash imaging (**middle**) and myocardial replenishment on the **right**, where complete and homogeneous myocardial perfusion can be observed. Sequences of myocardial replenishement were acquired at baseline, during coronary occlusion, after 30 minutes of reperfusion and with adenosine infusion.

Myocardial contrast echocardiography was performed with a real-time ultrasound system (HDI 5000 – Philips Medical Systems) equipped with a 4-2 MHz transducer. Images were acquired in the longitudinal apical view by using a water-filled latex interface between anterior epicardium and the transducer. Power Pulse Inversion imaging was adjusted before contrast injection to minimize the clutter artifact due to cardiac motion. Specific instrumentation settings included: mechanical index ranging from 0.09 to 0.12 (usually 0.1), low dynamic range and maximal line density. After adjustment, all parameters, including depth, focal point and gain, were held constant for each experiment. Manually triggered, transient, high mechanical index imaging ("Flash" imaging) was deflagrated at peak contrast intensity to destroy microbubbles within the myocardium, exclude artifacts and allow the evaluation of myocardial replenishment (Figure 1) [10].

The contrast agent used was Perfluorocarbon-Exposed Sonicated Dextrose Albumin (PESDA), prepared in our laboratory according to previous description [11]. Briefly, 8 ml of Decafluorobutane gas (Fluoromed Corporation, Round Rock, Texas, USA) were hand agitated with a 3:1 mixture of 5% dextrose and 5% human serum albumin. This mixture was then sonicated for 80 seconds. The mean concentration of microbubbles produced was 1.4 × 10^9 ^microbubbles/ml with a mean size of 4.6 ± 0.2 microns. A total of 0.1 ml/Kg of PESDA was diluted in 80 ml of 0.9% saline and administered by continuous intravenous infusion. The infusion was increased to a rate of 1–3 ml·min^-1^, and adjusted so that there was good opacification of left ventricular cavity and adequate myocardial perfusion imaging.

### Experimental protocol

Baseline RTMCE was obtained when hemodynamic stability had been achieved. LAD was then totally occluded for 180 minutes, with confirmation of absent coronary flow by electromagnetic flowmeter with continuous monitoring of hemodynamics. The occluder was then withdrawn and, at 30 minutes of reperfusion, myocardial wall motion (WM) and perfusion was analysed using RTMCE once coronary flow had reached a constant rate. Image acquisition was performed before and after a four-minutes intravenous infusion of adenosine at a rate of 140 mcg·Kg^-1^·min^-1^, using a infusion pump.

### Imaging analysis

The complete sequence of flash imaging with high energy followed by subsequent myocardial refilling was acquired and stored on VHS videotape and optical disk for off-line processing and analysis. Sequences were acquired at reperfusion and compared to baseline to identify perfusional abnormalities.

IS was defined by RTMCE as the residual perfusional myocardial defect area after coronary reperfusion, before and during adenosine infusion, and compared to tissue staining [12]. At the moment of maximal myocardial contrast replenishment, the borders of perfusion defects were visually identified as the largest area with clearly diminished opacification, and measured by digital planimetry, in a single end-diastolic frame.

Blood pressure, heart rate and LAD flow were collected at baseline, at 180 minutes of occlusion, at 30 minutes of reperfusion and during adenosine. IS was determined by two observers blinded of tissue staining measurements in all dogs.

### Hystological staining

After completion of the protocol, animals were euthanized and had their heart excised. The LAD was again occluded at the same site and cannulated immediately after occlusion. Ostium of the right coronary artery and the left main branch were also cannulated. Evans blue dye (1 mg·Kg^-1^) was infused in the right coronary artery and left main branch and, simultaneously, a 2% solution of 2.3.5-triphenyl-tetrazolium chloride (TTC) was injected into LAD, during 5 minutes. The heart was then sectioned longitudinally, passing through aortic valve and cardiac apex and incubated into TTC at 37°C for 30 minutes. This technique stains viable myocardium brick red and spares necrotic tissue. Regions that failed to demonstrate brick red staining, appearing pale yellow, were considered necrotic myocardium [13]. Evans blue stained of blue the regions not perfused by LAD and, therefore, defined the risk area. The TTC-stained slice correspondent to that of echocardiographic apical longitudinal view was digitalized into an off-line computer and the necrotic area was calculated by planimetry.

### Statistical analysis

Continuous and normally distributed data were expressed as mean ± one standard deviation (SD) and qualitative data as proportions. Within-groups comparisons were performed by paired Student's *t *test, repeated-measures analysis of variance or Friedman's test, as appropriate. Correlations between the RTMCE before and during adenosine and tissue staining were performed by linear regression using Spearman's rank statistic and agreement analysis as proposed by Bland and Altman. A p value < 0.05 (two-sided) was considered statistically significant.

## Results

Three dogs had ventricular fibrillation and were excluded from the study. The protocol was successfully performed in the remaining nine dogs (mean weight of 15.8 ± 2.3 Kg). Technically adequate contrast imaging was obtained in all animals.

### Hemodynamic data

Hemodynamic data are summarized in Table 1. Mean arterial blood pressure and heart rate remained stable during the entire occlusion and reperfusion stages. The epicardial LAD flow reduced to zero during LAD occlusion. In the first minute of reperfusion, we observed a period of reactive hyperemia with an increase of 2.6 times (54.3 ± 25.6 ml·min^-1^) the baseline LAD flow. At 30 minutes of reperfusion, LAD flow had already returned to baseline levels. Intravenous infusion of PESDA caused no significant changes in baseline hemodynamic values.

**Table 1:** Hemodynamic data at baseline, at 180 minutes of coronary occlusion and at 30 minutes of reperfusion, before and during adenosine infusion.

	Baseline	LAD Occlusion	Reperfusion	Reperfusion + adenosine
HR (beats·min^-1^)	124 ± 12	112 ± 24	107 ± 24	106 ± 23
Mean ABP (mmHg)	87 ± 15	87 ± 19	78 ± 19	77 ± 18
LAD Flow (ml·min^-1^)	21.1 ± 5.3	------	21.2 ± 5.7*	19.9 ± 6.0†

Values are mean ± SD. ABP = arterial blood pressure, LAD = left anterior descending coronary artery, HR = heart rate. * p = NS between baseline and reperfusion. † p < 0.05 between baseline and reperfusion + adenosine.

### Determination of IS by RTMCE and tissue staining

**Figure 2:**
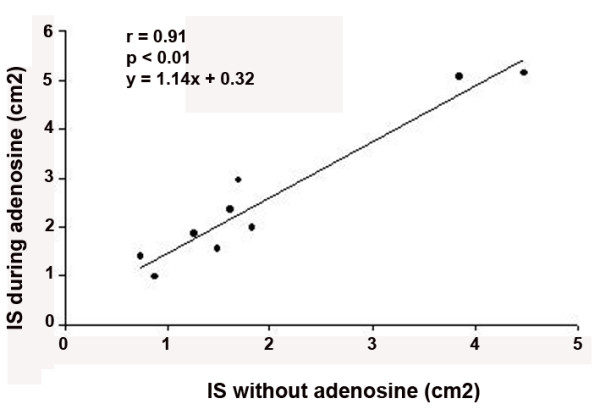
Correlation between infarct size (IS) determined by real-time myocardial contrast echocardiography before and during adenosine infusion.

**Figure 3:**
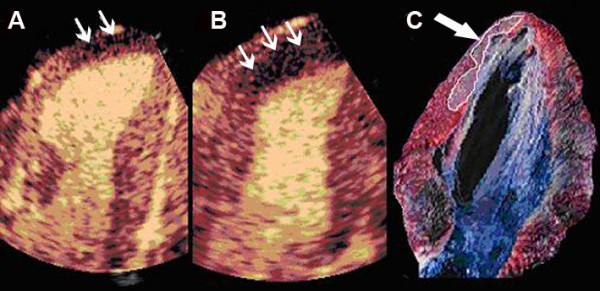
Representative example of real-time myocardial contrast echocardiography images showing lack of perfusion that corresponds to infarcted area before **(A) **and during adenosine infusion (small arrows) **(B)**. Note that adenosine increases the infarct size determination. Necrotic area was determined as the region that failed to demonstrate brick red staining, appearing pale yellow, by triphenyl-tetrazolium chloride staining (arrow) **(C)**.

At baseline, both left ventricular wall thickening and myocardial perfusion were normal in all dogs. We observed wall motion abnormalities and perfusional defects in the area supplied by LAD soon after coronary occlusion. IS determined by RTMCE 30 minutes after coronary reperfusion was 1.98 ± 1.30 cm^2^, and increased to 2.58 ± 1.53 cm^2 ^during adenosine infusion (p = 0.004). Adenosine consistently resulted in a slightly greater area of IS (see Table 2 for details), with a strong correlation of measurements before and after its use (Figure 2). Infarcted area was localized mostly in the subendocardium, inside the region supplied by LAD, and in the correspondent area of necrotic tissue as determined by TTC staining (Figure 3).

**Table 2:** Infarct size area determined by real-time myocardial contrast echocardiography (RTMCE) before and during adenosine infusion, and by triphenyl-tetrazolium chloride (TTC) staining

	Infarct Size (cm^2^)		
	RTMCE Before Adenosine	RTMCE During Adenosine	TTC staining

Dog 1	0.88	0.98	0.93
Dog 2	1.49	1.54	1.51
Dog 3	1.83	1.97	1.97
Dog 4	1.26	1.86	1.78
Dog 5	1.62	2.36	2.18
Dog 6	4.48	5.14	4.84
Dog 7	3.85	5.05	4.16
Dog 8	0.74	1.38	1.08
Dog 9	1.70	2.95	3.02

**Figure 4:**
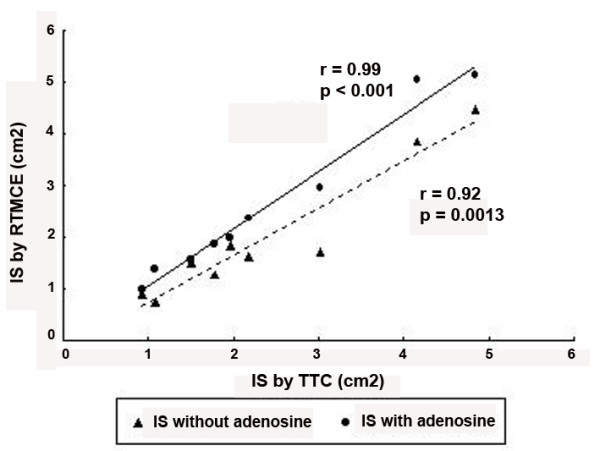
Correlation between infarct size (IS) determined by real-time myocardial contrast echocardiography (RTMCE) without adenosine (**triangles**) and during adenosine infusion (**circles**) and by triphenyl-tetrazolium chloride (TTC) staining.

**Figure 5:**
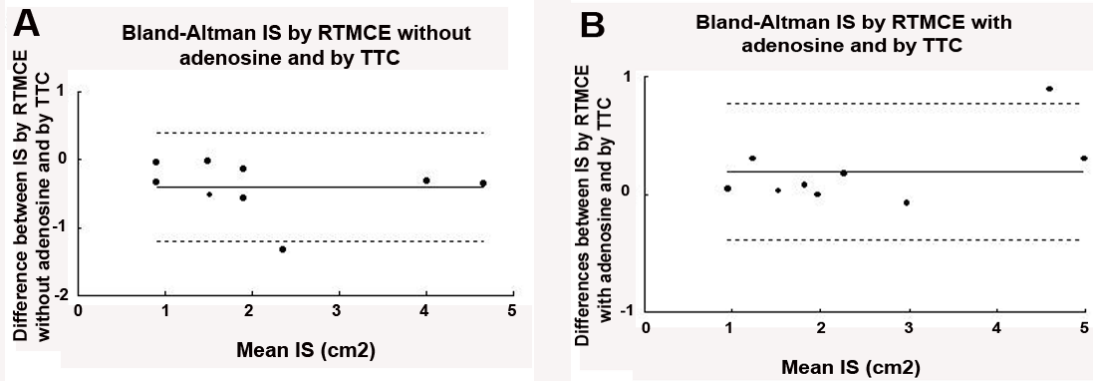
Bland-Altman plots showing mean difference (solid line) and limits of agreement (dashed lines) between infarct size (IS) determined by triphenyl-tetrazolium (TTC) staining and real-time myocardial contrast echocardiography (RTMCE) without adenosine **(A) **and during adenosine infusion **(B)**.

The necrotic myocardial area determined by TTC staining was 2.29 ± 1.36 cm^2^. There was nor a significant difference between IS determined by RTMCE before adenosine and TTC (p = 0.53) or during adenosine and TTC (p = 0.78). There was a strong correlation between RTMCE and TTC measurements both before (IS by TTC = 1.00 × IS by RTMCE + 0.40; r = 0.92, p = 0.0013) and during adenosine infusion (IS by TTC = 0.87 × IS by RTMCE with adenosine + 0.13; r = 0.99, p < 0.001), as demonstrated in Figure 4. However, a better concordance between the two measurements was observed with the use of exogenous vasodilator (Figure 5).

### Interobserver variability

Measurements of IS evaluated by the two independent observers showed a good concordance. Before adenosine infusion, the interobserver variability, expressed by the relative mean error, was 2.7% whereas during adenosine infusion it was 9.4%.

## Discussion

Although some studies have already evaluated the use of RTMCE for assessment of myocardial perfusion after acute myocardial infarction [14], the role of exogenous vasodilatation in this setting is not well established. Our study confirmed previously described findings reporting that, in experimental model of infarction, RTMCE is capable of determining IS accurately after coronary reperfusion, instead of disparity between abnormal WM and IS at rest and perfusional defect during demand ischemia that was explained by the presence of collateral blood flow [15]. During adenosine infusion, we observed an increase in perfusional defect, resulting in a better correlation between the echocardiographic measurements and the necrotic area determined by tissue staining. Actually, adenosine infusion unmasked a very small region of infarcted myocardium, which was surrounding the central necrotic tissue area.

It is well known that in the immediate period after acute infarction, myocardium within the reperfusion to the ischemic bed is variable. Irreversibly injured myocardium is generally associated with loss of microvascular integrity, which is proportional to the extension and severity of myocellular necrosis [16]. However, because of the wave-front nature of myocardial damage following a prolonged period of ischemia, a mismatch between microvascular and myocyte necrosis can occur, resulting in areas of cellular death with relatively preserved perfusion [1]. In the poorly perfused areas, severe capillary damage and coagulation necrosis can be observed, but microvascular damage might also develop during reperfusion. Due to its ability to remain entirely within intravascular space, microbubbles used as echocardiographic contrast agents are markers of blood flow and allows a non-invasive evaluation of microvascular integrity. Previous studies of myocardial contrast echocardiography have shown that, after infarct-related artery reperfusion, the spatial distribution of myocardial perfusional defects varies with time and by itself can underestimate the degree of necrosis. In these cases, regions within infarcted area with preserved microvasculature but depressed flow reserve can be unmasked by infusion of an exogenous vasodilator [5]. On the other hand, it was also previously demonstrated in experimental studies, that RTMCE can accurately delineate risk area and IS after reperfusion. With this technique, the size of an opacification defect varies with the period of time available for myocardial refilling by microbubbles [12]. Therefore, the infarct area should be determined at the end of the myocardial refilling sequence.

Our findings are that the analysis of myocardial perfusion by RTMCE slightly underestimated the IS, while the addition of adenosine resulted in a better correlation with TTC. However, we would like to emphasize that correlation between RTMCE and TTC was good even before adenosine infusion. Some mechanisms should be considered when analyzing possible factors related to the better IS determination using RTMCE than previously related reports using intermittent imaging techniques [3]. It is important to note that the combination of intravenous continuous infusion of a perfluorocarbon-based microbubble and a low energy imaging modality allow infusion of small quantities of contrast agent, avoiding over-saturation of microbubbles and reducing blooming effects. In contrast, some initial studies used intraaortic injection of air-filled microbubbles as echocardiographic agent, resulting in a higher concentration of microbubbles in the microcirculation, and, perhaps, leading to an over saturation of contrast [3,6]. Another reasonable associated mechanism for these findings would be the longer interval required for each acquisition when using intermittent imaging. In this modality of imaging, ultrasound pulses are emitted intermittently and triggered by electrocardiogram, and this longer period of time between imaging could allow the opacification of areas with infarcted myocardium but still preserved microcirculation. In this context, the benefits of vasodilator use have already been established since they unmask dysfunctional microvasculature. In the current study we analyzed the destruction/refilling curves derived from low-energy real-time imaging. It seems that this technique provides a good determination of myocardial perfusion, even without vasodilator use.

Magnetic resonance imaging with gadolinium-based contrast was been demonstrated a precise method for determining IS and its transmurality [17,18]. Magnetic resonance imaging offers high spatial resolution and can identify non-viable tissue using delayed enhancement technique. However, its cost and availability limit the widespread use of this technique. RTMCE is a non-radiologic modality of imaging that permits the evaluation of patients at bedside, and has the advantage of providing rapid information regarding myocardial contraction and perfusion. Strain rate imaging is another echocardiographic imaging that has been recently shown useful for differentiating transmural from non-transmural myocardial infarction in a small number of patients [19].

### Limitations

IS estimation has been demonstrated limited immediately after reperfusion because of reactive hyperemia that occurs shortly after reperfusion and abates within hours [20]. Previous studies have demonstrated that myocardial contrast echocardiography with intermittent harmonic imaging performed after twelve hours of reperfusion provides an accurate assessment of infarct size [21]. One could argue that the period in which myocardial perfusion was evaluated in our study was too close after LAD reperfusion (30 min). However, the study was designed to determine the value of RTMCE in a period of time in which reactive hyperemia is known to occur [20].

The assessment of IS by two-dimensional contrast echocardiography was performed using a single tomographic plane. Consequently, the measurements obtained by RTMCE and tissue staining represent an estimation of the true spatial extent of infarcted myocardium. However, since the aim of this study was a direct comparison between IS determined before and during adenosine with anatomical area, this issue is minimized. Of note, previous animal studies validated the good agreement between echocardiographic measurements and anatomic findings of risk area as well as IS using the methodology employed in this study [22,23].

Our results are based on visual assessment of perfusion defects and their measurements by planimetry. Although myocardial blood flow quantification can provide information about myocardial viability after reperfusional therapy, we considered that it was not necessary for the design of this study.

### Clinical implications

A prompt definition of the efficacy of reperfusion therapy as well as the extent of its residual microvascular damage by a non-invasive method has important implications for the management of patients with acute myocardial infarction. Also, the presence of preserved microvascular flow in the post-reperfusion period is associated with a lower rate of fibrous scar and less ventricular remodeling [2,24]. Our study showed that RTMCE can provide a reliable estimative of IS by visual evaluation, even without the infusion of exogenous vasodilator. This seems to be an important contribution for the clinical application of myocardial contrast echocardiography in the emergency room to delineate IS in the setting of acute myocardial infarction, since some patients may present in a hemodynamically unstable period after reperfusional therapy, precluding the use of adenosine.

## Competing interests

The author(s) declare that they have no competing interests.

## Authors' contributions

PMMD and WMJ designed the study and wrote the manuscript. JMT and JCNS performed echocardiographic examinations and provided input into manuscript revisions. VDA performed histological analysis. ACPC, PLL and JAFR provided knowledge into the myocardial infarction experimental model and performed manuscript review, with important intellectual contents.

## References

[B1] Reimer KA, Jennings RB (1979). The "wavefront phenomenon" of myocardial ischemic cell death. II. Transmural progression of necrosis within the framework of ischemic bed size (myocardium at risk) and collateral flow. Lab Invest.

[B2] Ito H, Maruyama A, Iwakura K, Takiuchi S, Masuyama T, Hori M, Higashino Y, Fujii K, Minamino T (1996). Clinical implications of the 'no reflow' phenomenon. A predictor of complications and left ventricular remodeling in reperfused anterior wall myocardial infarction. Circulation.

[B3] Villanueva FS, Glasheen WP, Sklenar J, Kaul S (1993). Characterization of spatial patterns of flow within the reperfused myocardium by myocardial contrast echocardiography. Implications in determining extent of myocardial salvage. Circulation.

[B4] Kloner RA, Ganote CE, Jennings RB (1974). The "no-reflow" phenomenon after temporary coronary occlusion in the dog. J Clin Invest.

[B5] Kaul S (1997). Myocardial contrast echocardiography: 15 years of research and development. Circulation.

[B6] Kaul S, Villanueva FS (1992). Is the determination of myocardial perfusion necessary to evaluate the success of reperfusion when the infarct-related artery is open?. Circulation.

[B7] Vanhaecke J, Flameng W, Borgers M, Jang IK, Van de WF, De GH (1990). Evidence for decreased coronary flow reserve in viable postischemic myocardium. Circ Res.

[B8] Johnson WB, Malone SA, Pantely GA, Anselone CG, Bristow JD (1988). No reflow and extent of infarction during maximal vasodilation in the porcine heart. Circulation.

[B9] Porter TR, Xie F, Silver M, Kricsfeld D, Oleary E (2001). Real-time perfusion imaging with low mechanical index pulse inversion Doppler imaging. J Am Coll Cardiol.

[B10] Pelberg RA, Wei K, Kamiyama N, Sklenar J, Bin J, Kaul S (1999). Potential advantage of flash echocardiography for digital subtraction of B-mode images acquired during myocardial contrast echocardiography. J Am Soc Echocardiogr.

[B11] Porter TR, Xie F (1995). Transient myocardial contrast after initial exposure to diagnostic ultrasound pressures with minute doses of intravenously injected microbubbles. Demonstration and potential mechanisms. Circulation.

[B12] Jayaweera AR, Matthew TL, Sklenar J, Spotnitz WD, Watson DD, Kaul S (1990). Method for the quantitation of myocardial perfusion during myocardial contrast two-dimensional echocardiography. J Am Soc Echocardiogr.

[B13] Fishbein MC, Meerbaum S, Rit J, Lando U, Kanmatsuse K, Mercier JC, Corday E, Ganz W (1981). Early phase acute myocardial infarct size quantification: validation of the triphenyl tetrazolium chloride tissue enzyme staining technique. Am Heart J.

[B14] Lafitte S, Higashiyama A, Masugata H, Peters B, Strachan M, Kwan OL, DeMaria AN (2002). Contrast echocardiography can assess risk area and infarct size during coronary occlusion and reperfusion: experimental validation. J Am Coll Cardiol.

[B15] Leong-Poi H, Coggins MP, Sklenar J, Jayaweera AR, Wang XQ, Kaul S (2005). Role of collateral blood flow in the apparent disparity between the extent of abnormal wall thickening and perfusion defect size during acute myocardial infarction and demand ischemia. J Am Coll Cardiol.

[B16] Kloner RA, Rude RE, Carlson N, Maroko PR, DeBoer LW, Braunwald E (1980). Ultrastructural evidence of microvascular damage and myocardial cell injury after coronary artery occlusion: which comes first?. Circulation.

[B17] Simonetti OP, Kim RJ, Fieno DS, Hillenbrand HB, Wu E, Bundy JM, Finn JP, Judd RM (2001). An improved MR imaging technique for the visualization of myocardial infarction. Radiology.

[B18] Mahrholdt H, Wagner A, Judd RM, Sechtem U (2002). Assessment of myocardial viability by cardiovascular magnetic resonance imaging. Eur Heart J.

[B19] Zhang Y, Chan AK, Yu CM, Yip GW, Fung JW, Lam WW, So NM, Wang M, Wu EB, Wong JT, Sanderson JE (2005). Strain rate imaging differentiates transmural from non-transmural myocardial infarction: a validation study using delayed-enhancement magnetic resonance imaging. J Am Coll Cardiol.

[B20] Kaul S (1999). Myocardial contrast echocardiography in acute myocardial infarction: time to test for routine clinical use?. Heart.

[B21] Ragosta M, Camarano G, Kaul S, Powers ER, Sarembock IJ, Gimple LW (1994). Microvascular integrity indicates myocellular viability in patients with recent myocardial infarction. New insights using myocardial contrast echocardiography. Circulation.

[B22] Grayburn PA, Erickson JM, Escobar J, Womack L, Velasco CE (1995). Peripheral intravenous myocardial contrast echocardiography using a 2% dodecafluoropentane emulsion: identification of myocardial risk area and infarct size in the canine model of ischemia. J Am Coll Cardiol.

[B23] Kaul S, Glasheen W, Ruddy TD, Pandian NG, Weyman AE, Okada RD (1987). The importance of defining left ventricular area at risk in vivo during acute myocardial infarction: an experimental evaluation with myocardial contrast two-dimensional echocardiography. Circulation.

[B24] Agati L, Voci P, Bilotta F, Luongo R, Autore C, Penco M, Iacoboni C, Fedele F, Dagianti A (1994). Influence of residual perfusion within the infarct zone on the natural history of left ventricular dysfunction after acute myocardial infarction: a myocardial contrast echocardiographic study. J Am Coll Cardiol.

